# Molecular epidemiology and drug resistance of *Acinetobacter baumannii* isolated from a regional hospital in the Brazilian Amazon region

**DOI:** 10.1590/0037-8682-0087-2020

**Published:** 2020-11-13

**Authors:** Edlainny Araujo Ribeiro, Ana Cristina Gales, Ana Paula Streling de Oliveira, Danilo Dias Coelho, Rodrigo Alves de Oliveira, Irmtraut Araci Hoffmann Pfrimer, José Rodrigues do Carmo

**Affiliations:** 1 Pontifícia Universidade Católica de Goiás, Programa de Pós-Graduação Stricto Sensu em Ciências Ambientais e Saúde, Goiânia, Goiás, Brasil.; 2 Universidade Federal de São Paulo, Laboratório Especial de Microbiologia Clínica, São Paulo, SP, Brasil.; 3 Hospital Regional Público do Araguaia, Redenção, PA, Brasil.

**Keywords:** Acinetobacter baumannii, Carbapenem resistance, Carbapenemase, *bla*_*OXA-23*_, Gram-negative bacilli

## Abstract

**INTRODUCTION:**

In this study, we report a clonal dissemination of carbapenem resistant *Acinetobacter baumannii* isolates due to the acquisition of bla_OXA-23_ in a regional hospital located in Brazilian Amazon Region.

**METHODS:**

The isolates were identified by MALDI-TOF and the carbapenemase-encoding genes were detected by multiplex-PCR. The genetic similarity was investigated by pulsed-field gel electrophoresis (PFGE).

**RESULTS:**

Only 10 (55.6%) isolates harbored the gene *bla*_OXA-23_. PFGE analysis revealed that these isolates belong to a single clone.

**CONCLUSIONS:**

This dissemination strategy indicates the need for surveillance, adoption of control procedures defined in guidelines, and the careful administration of antimicrobials should be reinforced.

*Acinetobacter baumannii* is an opportunistic pathogen with several virulence factors associated with several outbreaks worldwide, especially among intensive care unit (ICU) patients with severe underlying diseases^1^ ^,^ ^2^. These infections result in high mortality rates and increased treatment costs^1^ ^,^ ^2^. Approximately 700,000 deaths worldwide are associated with multidrug-resistant (MDR) microorganisms. By 2050, the estimated global economic losses could reach 60 to 100 trillion dollars if multidrug resistance is not controlled^3^. 

The worldwide emergence and dissemination of MDR bacteria, such as *A. baumannii*, led the World Health Organization (WHO) to gather global leaders at the United Nations (UN) General Assembly meeting in 2016 to commit them to fight against antimicrobial resistance^4^.

In infections caused by *A. baumannii*, genes encoding resistance to multiple broad-spectrum antimicrobials are commonly detected, including carbapenem resistance genes^5^. A study conducted by the SENTRY Antimicrobial Surveillance Program demonstrated that carbapenem resistance in Brazilian *A. baumannii* isolates increased by approximately 60% compared to resistance between the periods of 1977-1999 (12.6%) and 2008-2010 (71.4%)^6^.

In addition, it should be noted that environmental and patient colonization by MDR *A. baumannii* (MDR-AB) is a risk factor for the dissemination of this pathogen among patients and for the subsequent development of infections^7^. This bacterium remains viable for long periods in the environment, tolerates desiccation and is able to survive on inanimate dry surfaces for several months. All these conditions favor its rapid dissemination by cross-contamination in hospital environments^7^.

Hence, MDR-AB isolates are associated with high mortality rates, costs, and dissemination among hospitalized patients. Studying MDR-AB dissemination is important for implementing effective infection control/colonization measures, breaking the epidemiological chain of transmission of this microorganism, mitigating rates of bacterial resistance, reducing morbidity and mortality, and improving the quality of healthcare.

Considering these factors and the lack of research on this topic in the Brazilian Amazon region, the current study aimed to determine the phenotypic and genotypic characteristics of MDR-AB isolates, to detect the presence of carbapenemases, and to demonstrate the genetic similarity among carbapenemase-producing isolates at a tertiary referral hospital in the Amazon.

A cross-sectional descriptive study was performed between September 2017 and February 2018 at a regional public hospital in the southeast region of Pará state (Brazil). This is a tertiary referral hospital with different medical specialties and with pediatric and adult intensive care units, particularly for nephrology, with kidney transplantation and renal replacement therapy services. The hospital treats an estimated population of 541,000 inhabitants and is located in the Amazon biome. 

A total of 18 bacterial isolates were recovered from patients undergoing treatment at the regional hospital and diagnosed with a hospital-acquired infection (HAI) or with colonization caused by *A. baumannii* resistant to imipenem and meropenem were included in the study. Only one isolate per patient was analyzed. The isolates were obtained from clinical samples including tracheal secretions, blood cultures, postoperative wound swabs, catheter tips, and inguinal swabs. 

Antimicrobial susceptibility was performed using the disk diffusion test and the results were interpreted according to the Brazilian Committee on Antimicrobial Susceptibility Testing (BrCAST) guidelines^8^ for the following antimicrobials: piperacillin-tazobactam, ceftazidime, cefotaxime, cefepime, gentamicin, amikacin, tetracycline, ciprofloxacin, levofloxacin, trimethoprim sulfamethoxazole, imipenem, meropenem, and aztreonam. 

The minimum inhibitory concentration (MIC) of carbapenems was defined using the ETEST^®^ strip (bioMérieux) for isolates resistant to imipenem and also using the disk diffusion test for meropenem. MIC of polymyxin B (Sigma-Aldrich, St. Louis, MO, USA) was determined using the broth microdilution susceptibility test (Basingstoke, UK). The concentrations tested ranged from 0.125 to 64 µg/mL and the bacteria were considered resistant when the MIC values were ≥ 4 µg/mL^8^.

 MIC for tigecyclines was determined using the MicroScan AlkAway^®^ 96 plus (MIC panel type 40, Beckman Coulter, West Sacramento, USA) and interpreted according to the thresholds set by the Food and Drug Administration (FDA) (sensitive ≤2 µg/mL and resistant ≥8 µg/mL; https://www.accessdata.fda.gov/drugsatfda_docs). The standard strain *E. coli* ATCC 35218 was used as a control. The kits Carbapenembac™ and Carbapenembac-Metalo™ (PROBAC^®^) were used for phenotypic detection of carbapenemases and confirmation of positive isolates, respectively.

The identification of all positive isolates in the phenotypic test for carbapenemases (n = 10) was confirmed by MALDI-TOF. For genotyping the isolates, chromosomal DNA was extracted from 3-5 colonies after boiling for 15 min and centrifuged at 12,000 rpm at room temperature. PCR was performed in a final volume of 25 µL of master mix (TopTaq^®^- QIAGEN), containing 25 ng of DNA and 0.5 µL of each primer^9^. Primers for the following genes were used: *bla*_VIM_, *bla*_SPM_, *bla*_GIM_, *bla*_IMP_, *bla*_SIM_, *bla*_NDM_, *bla*_KPC_, *bla*_OXA-23_, *bla*_OXA-24_, *bla*_OXA-51_, *bla*_OXA-58_, and *bla*_OXA-143_ ^9^.

Genetic similarity between *A. baumannii* isolates was determined by pulsed-field gel electrophoresis (PFGE), using the molecular weight marker Lambda PFGE Ladder^®^ (GelSyringe ™). Isolates were sent to laboratory of Alerta at the Federal University of São Paulo (UFSP) for molecular typing. For this purpose, bacterial suspensions were digested with the restriction enzyme ApaI (Uniscience, Miami, USA) and the DNA fragments were separated by 1% agarose gel electrophoresis (Invitrogen, Eragny, France) in 0.5X TBE buffer (Tris base, boric acid and EDTA in distilled water). Electrophoresis was performed using the CHEF-DR II system (Bio-Rad Laboratories, California, USA) at 14 ºC, using 200 volts (6 V/cm) electric current with an initial switch time of 5 s and a final switch time of 35 s for 19 hrs. The gel was stained with UniSafe Dye^®^ (Uniscience, Miami, USA) and photographed under ultraviolet light. PFGE-stained photos and DNA fragments were examined using BioNumerics software version 5.0 (Applied Maths, Kortrijk, BE)^9^. The bands were automatically defined by the software and then individually checked by visual comparison. Data were interpreted according to the Sørensen-Dice coefficient^10^, and the dendrogram was constructed using the unweighted pair group method using arithmetic averages (UPGMA). Tolerance was set at 0.8%, and a similarity threshold of 80% was used to separate isolates into clonal clusters.

Samples were obtained from ICU patients (50%) and ward patients (50%). The isolates were collected from tracheal secretions (50%; n = 9), blood cultures (16.7%; n = 3), postoperative wound swabs (16.7%; n = 3), catheter tips (11.1%; n = 2), and inguinal swabs (5.6%; n = 1). Among the 18 isolates included in the study, the majority of patients were male (61.1%; n = 11), whose ages ranged from 18 to 86 years. The isolates were resistant to almost all antimicrobials tested, and remained susceptible to tigecycline and polymyxin B. As shown in Table1, one isolate was not tested for polymyxin B and tigecycline due to loss of cell viability during the susceptibility test (Table 1).

**TABLE 1:** Resistance pattern of *A. baumannii* (n = 18) isolates in hospitalized patients.

Isolates	MIC (µg/mL)				DISK DIFFUSION (mm)								
	POL	TIG	MER	IMP	PIT	CPM	TET	CAZ	CTX	CIP	GEN	AMI	ATM
C1	2	≤ 2	≥ 32	≥ 32	R	R	R	R	R	R	R	R	R
C2	2	< 1	≥ 32	≥ 32	R	R	R	R	R	R	R	R	R
C3	< 0.25	< 1	≥ 32	≥ 32	R	R	R	R	R	R	R	R	R
→C4 *	1	≤ 2	≥ 32	≥ 32	R	R	R	R	R	R	R	R	R
C5	0.50	< 1	≥ 32	≥ 32	R	R	R	R	R	R	R	R	R
C6	0.50	< 1	≥ 32	≥ 32	R	R	R	R	R	R	R	R	R
C7	0.50	< 0.5	≥ 32	≥ 32	R	R	R	R	R	R	R	R	R
→C8**	1	≥ 8	≥ 32	≥ 32	R	R	R	R	R	R	R	R	R
→C9**	< 0.25	< 1	≥ 32	≥ 32	R	R	R	R	R	R	R	R	R
C10	0.50	< 1	≥ 32	≥ 32	R	R	R	R	R	R	R	R	R
→C11*	2	≥ 8	≥ 32	≥ 32	R	R	R	R	R	R	R	R	R
→C12**	NT	NT	≥ 32	≥ 32	R	R	R	R	R	R	R	R	R
→C13**	0.50	< 1	≥ 32	≥ 32	R	R	R	R	R	R	R	R	R
→C14*	2	< 1	≥ 32	≥ 32	R	R	R	R	R	R	R	R	R
C15	0.50	< 1	≥ 32	≥ 32	R	R	R	R	R	R	R	R	R
→C16**	0.50	< 1	≥ 32	≥ 32	R	R	R	R	R	R	R	R	R
→C17**	< 0.25	≥ 8	≥ 32	≥ 32	R	R	R	R	R	R	R	R	R
→C18**	1	< 1	≥ 32	≥ 32	R	R	R	R	R	R	R	R	R

**POL:** polymyxin; **AMI:** amikacin; **TGC:** tigecycline; **ATM:** aztreonam; **CPM:** cefepime; **CAZ:** ceftazidime; **CTX:** cefotaxime; **CIP:** ciprofloxacin; **GEN:** gentamicin; **IMP:** imipenem; **MER:** meropenem; **PIT:** piperacillin-tazobactam; **TET:** tetracycline; **R:** resistant; **S:** sensitive; **NT:** not tested. *Isolate phenotypically positive for metalo-β-lactamase; **Isolate phenotypically positive for serino-carbapenemases; → Isolates positive for *bla* ^OXA-23^ and *bla* ^OXA-51^.

The phenotypic assay showed that all isolates were MDR bacteria, including 10 (55.6%) which were carbapenemase-producing. Three of them (16.7%) were positive for metalo-β-lactamases and seven (83.3%) for serine carbapenemases. However, genotypic testing did not confirm these findings (Table 1). In all 10 isolates characterized as carbapenemase-producing, we found the presence of *bla*_OXA-23_ and *bla*_OXA-51_ gene. PFGE analysis showed that all isolates which tested positive for carbapenemase belonged to a single clone (Figure 1).

**FIGURE 1:**
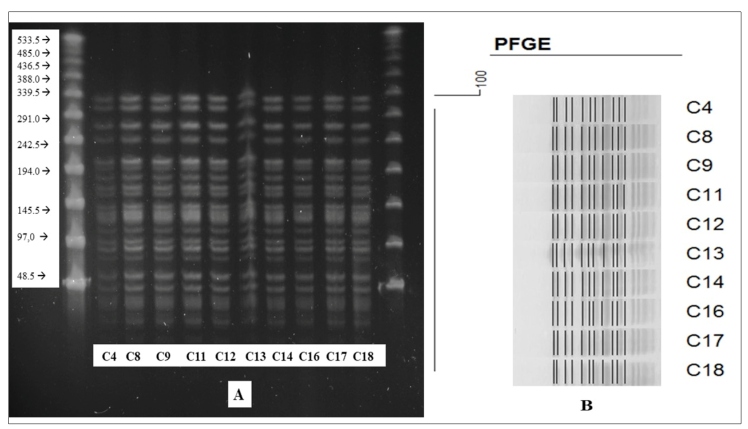
A) PFGE demonstrating similarity among carbapenemase-producing isolates positive for *bla-OXA-23*. B) A dendrogram representing PFGE profiles of carbapenemase-producing *A. baumannii* isolates from 10 patients undergoing treatment at a Brazilian Amazon hospital. The identification number of the isolates is found to the right of the profiles.

MDR-AB is more frequently isolated from colonized and/or HAI patients, especially in hospital environments^2^ ^-^ ^4^. These infections occur mainly in patients with severe underlying disease and poor prognosis who are treated with invasive procedures, using broad-spectrum antibiotics, and are admitted to the ICU^2^ ^-^ ^4^. Treating infections caused by MDR-AB is complicated, especially when these bacteria are resistant to all antimicrobials commonly used in clinical practice^11^. In the present study, all isolates showed a multi-resistance profile, and some showed similar polymyxin B sensitivity rates. This is likely due to extensive and/or inadequate use of these antimicrobials in the treatment of infections caused by gram-negative MDR bacteria^11^. A study conducted in Rio de Janeiro, Brazil, demonstrated that most isolates (81.5%) were resistant to polymyxins, highlighting the importance of using antimicrobials adequately^11^.

In this study, most of the isolates were susceptible to tigecycline. Although this antimicrobial has been licensed for treating complicated intra-abdominal and skin infections and community-based bacterial pneumonia, it has been widely used off-label to treat many other infections, including those caused by MDR-AB^12^. However, treatment should be individualized and defined based on the best evidence obtained for combinatorial approaches^12^.

Most *A. baumannii* isolates in this study were collected from colonized ICU patients. It should be noted that either patient colonization or infection with MDR-AB are important sources of dissemination of resistant strains between hospitals^12^. One such example was the intercontinental transfer of patients colonized by MDR-AB after repatriation from Tahiti, resulting in a prolonged outbreak^7^. 

The genes *bla*_OXA-23_ and *bla*_OXA-51_ were detected in all carbapenemase-producing isolates, similar to what has been described in a previous study, which reported a carbapenem resistance rate of 90%^13^. These genes encode the most common OXA-type carbapenemases that contribute to resistance to imipenem and meropenem in *A. baumannii* isolates that are endemic in several Brazilian states^13^. 

The oxacillinase (OXA)-type carbapenemase expression is the most common mechanism of resistance in *A. baumannii*, but less effective than other enzymatic mechanisms^13^. Nevertheless, oxacillinase-producing isolates are known to be MDR bacteria. Microorganisms expressing bla_OXA-23_ have high MICs for imipenem and meropenem, but those expressing only *bla*_OXA-51_ have a lower MIC due to the reduced hydrolytic activity of OXA-51 for carbapenens^5^ ^,^ ^13^. 

The PFGE pattern demonstrated a high genetic similarity and dissemination of *bla*_OXA-23_-encoding *A. baumannii* strains, matching previous studies on ICU isolates which reported 91.8% and 100% of genetic identity^14^ ^,^ ^15^. This PFGE pattern suggests cross-contamination of *A. baumannii* isolates related to patient infection or colonization, whose source of dissemination could have been health staff, equipment, or contaminated fomites^14^. Ward patients included in the study had been previously admitted to the ICU, which suggests that the ICU was the primary source of cross-contamination of MDR-AB^14^ ^,^ ^15^.

Preventing the clonal dissemination of microorganisms and improvement in the healthcare quality requires strategic prevention efforts and well-known infection control practices. For example, applying hand hygiene and equipment cleaning rules and creating staff awareness are particularly important to prevent the spread of an infection^14^ ^,^ ^15^.

The results of this study demonstrate that carbapenem resistance was common to all *A. baumannii* isolates studied, and that all isolates had MDR bacteria. The genes *bla*_OXA-23_ and *bla*_OXA-51_ were detected in all carbapenemase-producing isolates, and all these isolates belonged to the same clone. Of all the antimicrobials tested, polymyxin B and tigecycline were the most effective antimicrobials for MDR-AB. The limitations identified in our study were the relatively small number of isolates from patients, who had several comorbidities and some of the patients were hospitalized multiple times; and the limited quality of the clinical data, which was insufficient to meet the study aims. 

Bacterial resistance is an emerging problem requiring the utmost attention and effort towards its mitigation. Our study highlights the need for screening colonized or infected patients and for providing frequent training to healthcare professionals in both ICUs and clinics. Moreover, surveillance for imipenem- and meropenem-resistant *A. baumannii* and rational administration of antimicrobials should be reinforced.
